# Surgical excision of giant vulvar angiofibroma: A case report and a review of literature

**DOI:** 10.1097/MD.0000000000030125

**Published:** 2022-09-09

**Authors:** Omar F. Altal, Shireen Rawashdeh, Sarah Al Sharie, Yazan O. Al Zu’bi, Ahmed H. Al Sharie, Majd N. Daoud, Khaled M. Alkhawaldeh

**Affiliations:** a Department of Obstetrics & Gynecology, Faculty of Medicine, Jordan University of Science and Technology, King Abdullah University Hospital, Irbid, Jordan; b Ministry of Health, Amman, Jordan; c Faculty of Medicine, Yarmouk University, Irbid, Jordan; d Faculty of Medicine, Jordan University of Science and Technology, Irbid, Jordan.

**Keywords:** benign vulvar neoplasms, cellular angiofibroma, soft tissue neoplasms

## Abstract

Cellular angiofibroma (CA) is a rare tumor of the soft tissue classified as a benign fibroblastic/myofibroblastic tumor. Considering this, the literature regarding CA mainly, but not exclusively, comprises single case reports and case series. Here, we report a case of giant CA of the vulva with comprehensive literature review.

**Patient concerns:**

We present a case of a massive vulvar CA arising in 53-year-old woman with no notable medical or surgical history. The mass has grown considerably over time, causing pain and difficult urination, defecation, and movement. The patient had normal regular menstrual cycle with no previous contraception use. Vaginal examination exposed a right-sided large tender vulvar mass with normal-looking vagina.

**Diagnoses:**

Pelvic magnetic resonance imaging with contrast revealed a large right vulvar heterogeneously enhancing soft tissue mass measuring 13.1 × 10.9 × 10.7 cm expending the left vulva, with internal and peripheral voids resembling feeding vessels. The mass was surgically removed, and subsequent histopathology showed skin-covered dermal-based lesion composed of fibroblast-like bland and spindle cell proliferation with thin-walled blood vessels of various sizes. Immunohistostaining of CD34 and smooth muscle antigen were both positive, while desmin was found to be negative. A diagnosis of vulvar angiofibroma was made based on the clinical scenario, imaging, and histopathology.

**Interventions:**

Mass vulvectomy was performed starting with a circumferential incision at the base of the mass and structural dissection to separate the mass from the vulvar wall. The incision was successfully closed, and subcuticular stitches were applied to the skin.

**Outcomes:**

The patient’s complaints were significantly relieved with no postoperative complications and the patient is being followed regularly in an outpatient setting.

**Lessons:**

Due to its extremely benign nature of CA, and the implausible ability of its recurrence, it was decided to surgically excise it. Despite its rarity, it can be readily identified at its earlier stages preventing the vexing and exasperating symptoms accompanied with increased size as mentioned.

## 1. Introduction

Benign vulvar masses arising in adult females encompass an expansive array of differential diagnoses. These range from cystic enlargement of any of the many anatomical glands of the distal female genital tract as well as a broad spectrum of noncancerous tumors of mesenchymal origin.^[1]^ Within this spectrum falls cellular angiofibroma (CA), to which attention has been drawn recently. CA is an uncommon, benign, soft tissue tumor that has equal incidence in both sexes. In women, it frequently occurs in the vulvovaginal region, while in men, it arises predominantly in the inguino-scrotal area. However, both extragenital and, to a lesser extent, extrapelvic occurrences have also been recounted.^[2]^ Normally, CA presents as a small, slowly growing, and painless mass within the bounds of superficial soft tissue. The agreed-upon surgical approach for treatment is complete local excision since it is very unlikely to recur and up to date has never traveled to distant sites.^[3]^ Herein we present a case of a uniquely large vulvar CA arising in a middle-aged woman with nonsignificant medical and surgical history. We supplement our case with a review of the literature on CA of the vulvovaginal area. This report was written in accordance with the CARE guidelines.^[4]^

## 2. Case presentation

**Figure 1.**
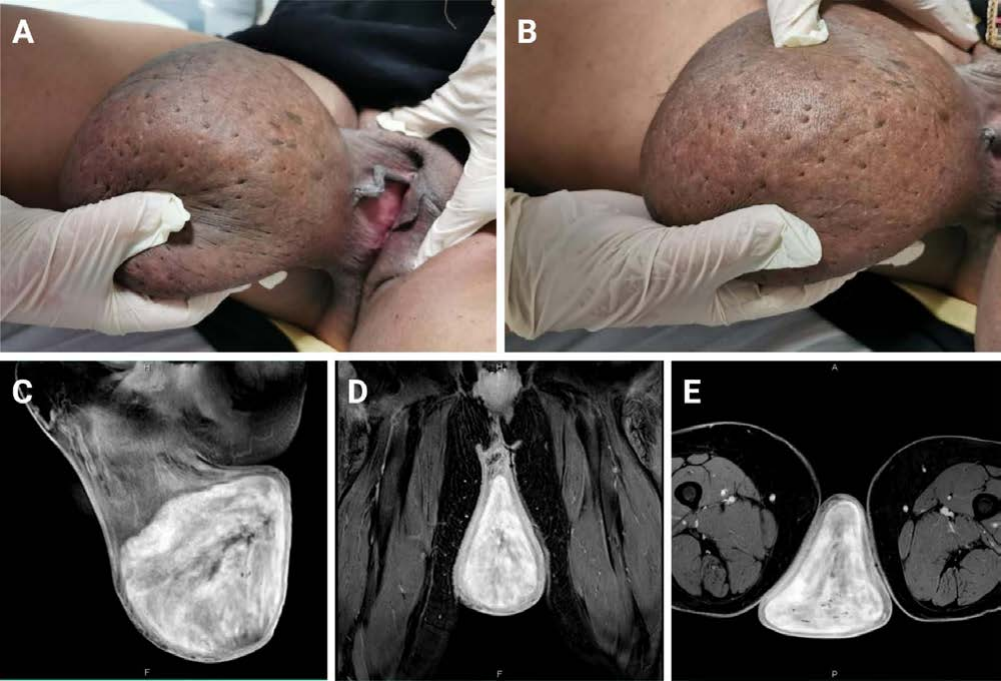
A right-sided large tender vulvar mass with normally looking vagina (A, B). Pelvic MRI with contrast revealing a large heterogeneously enhancing soft tissue mass, measuring 13.1 × 10.9 × 10.7 cm expending the left vulva with internal and peripheral voids resembling feeding vessels. MRI = magnetic resonance imaging.

**Figure 2.**
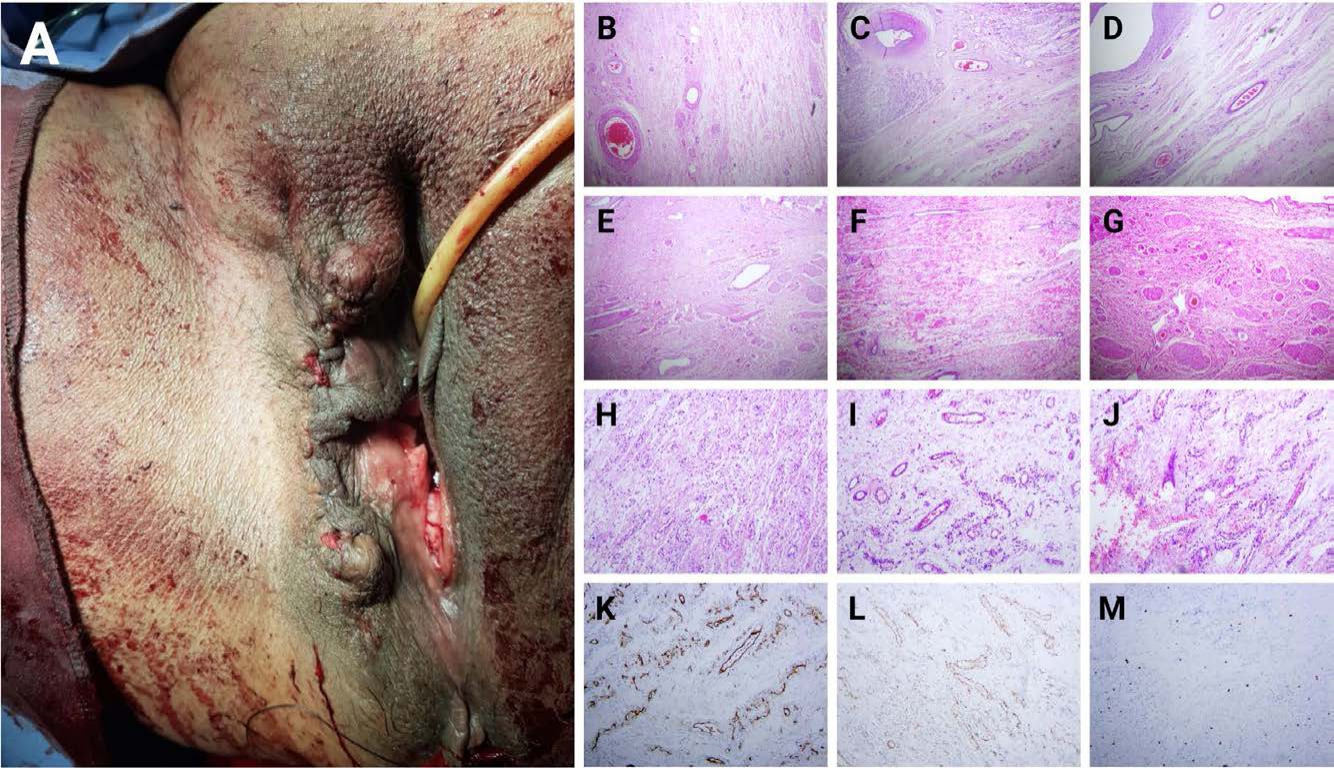
Mass vulvectomy was performed starting with a circumferential incision at the base of the mass and structural dissection to separate the mass from the vulvar wall. The incision was successfully closed, and subcuticular stitches were applied to the skin (A). Microscopic histopathological examination illustrated a skin-covered dermal-based lesion composed of fibroblast-like bland and spindle cell proliferation with variably sized thin-walled blood vessels and unremarkable adnexal structures (B–J). The cellular population was positive for CD34 (K) and SMA (L) but negative for desmin (M). SMA = smooth muscle antigen.

A 53-year-old women G7P5 + 2 with nonsignificant medical and surgical history presented to our gynecological outpatient’s clinic complaining of a giant vaginal mass. The mass was firstly noticed by the patient 9 months ago with progressive increase in size associated with pain and difficulty in urination, defecation, and movement, with no vaginal redness, itching, or bleeding. The patient reported a normal regular menstrual cycle occurring every 28 days and lasting 5 to 6 days each. In addition, no history of contraception or pap smears was reported. Vaginal examination revealed a right-sided large tender vulvar mass with normally looking vagina (**Fig. 1A**, B). Laboratory investigations including complete blood count, liver function test, kidney function test, urine analysis, and cancer markers were all within normal limits. Uterine ultrasonography was unremarkable. Pelvic magnetic resonance imaging with contrast was performed (**Fig. 1C–E**) to evaluate and assess the origin of the mass, which revealed a large heterogeneously enhancing soft tissue mass, measuring 13.1 × 10.9 × 10.7 cm expending the left vulva with internal and peripheral voids resembling feeding vessels. The mass was also associated with overlying subcutaneous edema and skin thickening. Incidental findings of intermural leiomyoma, few Nabothian cysts, and mild bilateral joint effusions with normally looking bilateral inguinal lymph nodes were noted. A decision was made to excise the mass. Under general anesthesia and in lithotomy position, a vulvectomy was performed, starting with a circumferential incision at the base of the mass and structural dissection to separate the mass from the vulvar wall. The incision was successfully closed, and subcuticular stitches were applied to the skin (**Fig. 2A**). Microscopic histopathological examination illustrated a skin-covered dermal-based lesion composed of fibroblast-like bland and spindle cell proliferation with variably sized thin-walled blood vessels and unremarkable adnexal structures (Fig. 2B–J). The cellular population was positive for CD34 and smooth muscle antigen but negative for desmin according to immunohistochemistry staining (Fig. 2K–M). The diagnosis was established as vulvar angiofibroma. The patient was discharged after 3 days following the operation and have been followed regularly with no reported complications.

## 3. Discussion

A systematic literature search was performed on 3 different databases (PubMed, Web of Science, and Scopus). Obtained records went through title and abstract screening, full-text screening, and data extraction by 2 independent authors (S.H and S.A; **Figure S1, Supplemental Digital Content**, http://links.lww.com/MD/H50). Case reports and case series describing CA were included. Irrelevant studies, those written in languages other than English, studies lacking full text and sufficient data, audits, and letters to editor were all excluded. Different parameters of the included case reports and case series were collected such as the age of the patient (years), site and size of the tumor (cm), clinical presentation, duration of symptoms (months), imaging modality, method of treatment, follow-up period (months), and histopathological findings. A summary of the included cases is demonstrated in Table 1.

**Table 1:** Baseline and disease characteristics of retrieved cases from the literature.

ID	Reference	Age (yr)	Site	Size (cm)	Duration (mo)	Clinical presentation	Imaging modality	Follow-up (mo)
1	Aydin et al 2016[12]	59	Right labia majora	20 × 15 × 10	60	Well-circumscribed mass	US, MRI	6
2	Kumar et al 2018[23]	38	Urethral meatus (right side)	1.9 × 2.9 × 2.7	96	Painless mass	US, MRI	24
3	Ahmadnia et al 2014[9]	20	Right labia majora	4.0 × 3.0 × 2.0	36	Painless mass	–	12
			Left labia majora	2.0 × 2.0 × 1.0				
4	McCluggage et al 2004[22]	49	Left labia majora	4.0, 6.5	–	–	–	33
		20	Not specified	2.4	–	–	–	240
		25	Vaginal introitus	1.0	–	–	–	3
		65	Left labia minora	5.0	–	–	–	12
		41	Left labia majora	2.0	–	–	–	4
		59	Vulva (right side)	2.0	–	–	–	18
		32	Right labia	1.5	–	–	–	–
5	Lane et al 2001[1]	77	Left labia	5.0	36	Asymptomatic mass	US	12
6	Curry et al 2001[24]	37	Clitoris	2.5	–	Vulvar neoplasm	US	15
7	Dargent et al 2003[25]	46	Labia	3.0 × 2.0 × 2.0	–	Painless mass	–	–
		49	Clitoris	4.0 × 2.0 × 2.0	–	Painless mass	–	–
8	Khmou et al 2016[26]	37	Left labia majora	3.0 × 3.0 × 2.5	72	Asymptomatic nodule	US	14
9	Micheletti et al 2005[27]	51	Right labia	7.0 × 5.5 × 1.4	24	Painless mass	–	–
10	Chen et al 2010[28]	58	Vulva	2.7	–	–	–	75
		52		3.0	–	–	–	27
		34		1.2	–	–	–	–
		32		2.7, 7.0	–	–	–	–
		25		1.3	–	–	–	42
		43		2.5	–	–	–	2
		59		1.3	–	–	–	14
		46		6.5	–	–	–	4
		71		7.5	–	–	–	–
		39		–	–	–	–	7
		46		2.0	–	–	–	–
11	Fachada et al 2020[23]	49	Vulva (right side)	5.5	24	Mass	–	10
12	Flucke et al 2011[29]	50	Vulva	3.0	–	–	–	55
		50		4.0	–	–	–	–
		48		8.5	–	–	–	–
		42		2.2	–	–	–	–
		42		1.7	–	–	–	30
		55		2.3	–	–	–	12
		57		4.5	–	–	–	6
		47		1.5	–	–	–	–
13	Priyadarshi et al 2012[30]	35	Right labia majora	8.0 × 6.0 × 4.0	36	Painless mass	–	32
		57	Left labia majora	6.0 × 6.0 × 5.0	24	Painless mass	–	24
14	Arsenovic et al 2009[31]	26	Vulvoinguinal area (right side)	12 × 8.5 × 5.0	7	Painless mass	–	24
15	Reder et al 2021[32]	62	Extended from the border of the mons pubis and the right portion of the vulva up to the knees	30 × 12 × 7.0	180	Asymptomatic mass	US	–
16	Kerkuta et al 2005[33]	31	Left labia majora	5.0 × 4.0 × 3.0	36	Painless mass	–	10
17	Current case	53	Vulva (right side)	13.1 × 10.9 × 10.7	9	Mass	MRI	–

MRI = magnetic resonance imaging, US = ultrasound.

CA is a relatively rare tumor. Considering this, the literature regarding CA mainly, but not exclusively, comprises single case reports and case series. The first description of CA was presented in 1997 by Nucci and colleagues. They described a series of vulval CAs in 6 middle-aged women and distinguished the lesion from angiomyofibroblastoma and spindle cell lipoma as a separate entity.^[5]^ A year later, Laskin et al presented 11 cases of a lesion very similar in its histology affecting adult men in the inguino-scrotal area. They used the term “angiomyofibroblastoma-like tumor” to describe it.^[6]^ In 2002, the World Health Organization added CA to the classification of soft tissue tumors under the category of benign fibroblastic/myofibroblastic tumors. Furthermore, any mass consisting of the typical histopathologic constituents was termed CA regardless of the sex of the patient it occurred in. This is due to the lack of morphologic differences between the sexes.^[7]^

CA has a predilection for the vulvovaginal region in females and the inguino-scrotal region in males. Despite the fact that CA occurs equally in both, it tends to arise earlier in women compared to men, during their 50s and 70s, respectively.^[2]^ Although the vast majority of reported CAs are confined to the pelvic area, a few unusual sites have been recounted. In 2015, Mandato and colleagues reported a case of a retroperitoneal CA causing the patient to present with coxalgia due to obturator nerve compression.^[8]^ The youngest report of vulvar CA was from a 20-year-old patient with additional lesions on the axilla and breast.^[9]^ In addition, the most recent peculiar site is CA of the orbit as described by Hötte et al.^[10]^

As for the clinical features of CA, patients usually present with a slowly-growing mass that is otherwise asymptomatic.^[3]^ Discomfort and difficulty moving may occur in lesions of the external genitalia that have reached a substantial size as in the case under study. The mass may be related to hernia or hydrocele in males.^[7]^ CA of the vulva is typically small in size (x̄ = 3.4 cm) and is almost always well circumscribed, whereas CAs in men tend to be larger in size (x̄ = 7.9 cm) and possess fewer clear margins.^[3,11]^ It is important to note that CA can continue to grow if left untreated. This is exemplified by our case as well as a case study by Aydin and colleagues in which they presented a vulvar CA that measured approximately 20 cm in width.^[12]^

Grossly, CAs appear as white or yellowish nodules that are firm and gelatinous in consistency. A cross-section through the lesions shows a white tan to grayish color. Their features of growth are mostly benign; they are usually well circumscribed and extension into neighboring soft tissue is scarcely seen.^[2]^ In the clinicopathologic and immunohistochemical analysis of 51 cases of CA by Iwasa et al, the majority of tumors resided in the superficial soft tissue while only a few cases had dermal involvement. None of the cases showed any evidence of necrosis but 1 case did exhibit foci of hemorrhage. Microscopically, cellular spindle cells and thick-walled stromal blood vessels are the 2 typical histologic components that make up a cellular angiofibroma. In many cases, peripheral clusters of adipocytes and fat lobules have also been reported.^[3]^

The immunohistochemical workup of CA is not present in all published studies. However, in cases where it was addressed, CA was found to be positive for CD34, smooth muscle actin, vimentin, and in over half of the cases, it also expressed estrogen and progesterone receptors. It is yet to be known whether this is of significance, considering the presence of these receptors is normal in the mesenchymal cells of the lower genital tract of females. Nonetheless, there are authors that hypothesize a role for estrogen and progesterone in CA pathogenesis. CA is desmin positive in only a minority of cases and is consistently negative for S100 protein and keratin.^[2,13]^

When contemplating the differential diagnoses of CA in women, there are numerous mesenchymal tumors that share close morphologic features. These include, spindle cell lipoma, solitary fibrous tumor, mammary-type myofibroblastoma, aggressive angiomyxoma (AA) and angiomyofibroblastoma. These are all typically composed of ovoid/spindle cells within a collagen stroma with thick-walled blood vessels of alternating size. Moreover, they all express immunoreactivity to CD34.^[2]^

The initial description of spindle cell lipoma, also known as pleomorphic lipoma, was in 1975 by Enzinger and Harvey.^[14]^ It is a specific type of lipoma that occurs almost exclusively in the shoulders, back, and posterior neck of adult males. However, a vulval lesion has been reported in the literature by Reis-Filho and colleagues.^[15]^ Both CA and spindle cell lipoma are positive for CD34 in 60% and 100% of the cases, respectively. The 2 can be differentiated by the fact that CAs have abundant, thick hyalinized blood vessels, whereas spindle cell lipomas have very thin-walled blood vessels and in general are unlikely to arise in the vulvovaginal region.^[7]^

Solitary fibrous tumor has been documented to appear in multiple anatomical sites, including the vulva and perineum.^[16,17]^ This tumor exhibits foci of hypercellular and hypocellular spindle cell growth in the background of keloidal collagen and branching hemangiopericytoma-like vessels.^[7]^ These alternating foci contrast solitary fibrous tumor to CA, wherein cellularity is more evenly distributed and more rounded small to medium-sized vessels are present.^[2]^

Mammary-type myofibroblastoma is a benign mesenchymal lesion with a propensity to arise along the recognized anatomic “milk-line.” It is most frequently seen in older men (male to female ratio is 8:2) in the inguinal/groin area.^[7]^ Grossly, is it a well-delineated nodular mass. Microscopically, it is composed of fascicles of myofibroblast-like spindle cells and variable amounts of adipose tissue, within a collagenous stroma. The tumor cells characteristically co-express CD34 and desmin. When comparing this tumor to CA, it is lacking the strikingly hyalinized vessels distinctive of CA. In addition, CA is almost always negative for desmin.^[18]^

AA is a locally recurring tumor of the vulvovaginal, perineal, and inguinal regions that is observed mainly in women ranging from 30 to 50 years old.^[19,20]^ AA has aggressive growth qualities that make it simple to distinguish from CA. It is large, deep-seated, and has an infiltrative growth pattern with entrapment of surrounding tissue. Under the microscope, AA has a lot less cells than CA and is unique in smooth muscle cells encircling blood vessels in a net-like motif.^[3]^

Angiomyofibroblastoma (AMF) and CA have numerous commonalities. AMF is a newly- described, benign growth that appears predominantly in the vulvovaginal region in women and occasionally in the inguino-scrotal region in men. It is well circumscribed and hence clinically mistaken as a Bartholin gland cyst.^[11]^ Upon histologic examination, AMF is found to comprised plump spindled and epithelioid or plasmacytoid mesenchymal cells that are distinctly woven around blood vessels and set in a myxedematous, loosely collagenous stroma.^[21]^ Intralesional adipose tissue and mast cells may be present.^[11]^ Immunohistochemistry proves useful in discerning AMF, as the cells are desmin positive and generally negative for CD34 and smooth muscle actin.^[2]^

Despite the restricted data, it is safe to say that CA acts in a benign manner. There is no mention in the literature of the lesion metastasizing and only 1 case of recurrence has been reported so far.^[2]^ In this study by McCluggage et al,^[22]^ the returned lesion was successfully excised and 33 months follow-up showed no signs of additional recurrence. A simple local excision with clean surgical margins is deemed sufficient for the management of these lesions.^[7]^

## 4. Conclusion

Herein, we reported a case of a massive vulvar angiofibroma, associated with pain and difficulty in urination, defecation, and movement. The mass grew over a period of 9 months, before the patient presented. Due to its extremely benign nature, and the implausible ability of its recurrence, it was decided to surgically excise it. Despite its rarity, it can be readily identified at its earlier stages preventing the vexing and exasperating symptoms accompanied with increased size as mentioned.

## Author contributions

Conceptualization: Omar F. Altal, Shireen Rawashdeh

Investigation: Omar F Altal. Shireen Rawashdeh

Methodology: Sarah Al Sharie, Yazan O. Al Zu’bi, Ahmed H. Al Sharie

Writing – original draft: Shireen Rawashdeh, Ahmed H. Al Sharie

Writing – review & editing: Majd N. Daoud, Khaled M. Alkhawaldeh

## Supplementary Material


