# Supplementary material for: Surgical excision of giant vulvar angiofibroma: A case report and a review of literature
Source: Medicine (Baltimore). 2022 Sep 9;101(36):e30125. doi: 10.1097/MD.0000000000030125 (PMC10980472; doi:10.1097/MD.0000000000030125)
Supplement: Supplementary file 1 [file medi-101-e30125-s001.pdf]

## Supplementary data

# Surgical Excision of Giant Vulvar Angiofibroma: A Case Report and a Review of Literature

Omar F. Altal[1*], Shireen Rawashdeh[2], Sarah Al Sharie[3], Yazan O. Al Zu'bi[4], Ahmed H. Al Sharie[4], Majd N. Daoud[4], Khaled M. Alkhawaldeh[2]

[1]Department of Obstetrics & Gynecology, Faculty of Medicine, Jordan University of Science and Technology, King Abdullah University Hospital, P. O. Box: 3030, Irbid, 22110, Jordan.

[2]Ministry of Health, Amman, Jordan.

[3]Faculty of Medicine, Yarmouk University, Irbid, 22110, Jordan.

[4]Faculty of Medicine, Jordan University of Science & Technology, Irbid, 22110, Jordan.

***Correspondence**:
Omar F. Altal
Department of Obstetrics and Gynecology, Faculty of Medicine
Jordan University of Science and Technology, King Abdullah University Hospital
P. O. Box: 3030, Irbid, 22110, Jordan.
Tel: +962797423400
Fax: +962 2 7201064
Email: altal_omar@yahoo.com

**Open Researcher and Contributor ID (ORCID):**
Sarah Al Sharie: 0000-0002-8004-0963
Ahmed H. Al Sharie: 0000-0003-1311-806X
Yazan O. Al Zu'bi: 0000-0002-2084-2374
Remaining authors don't have ORCID accounts.

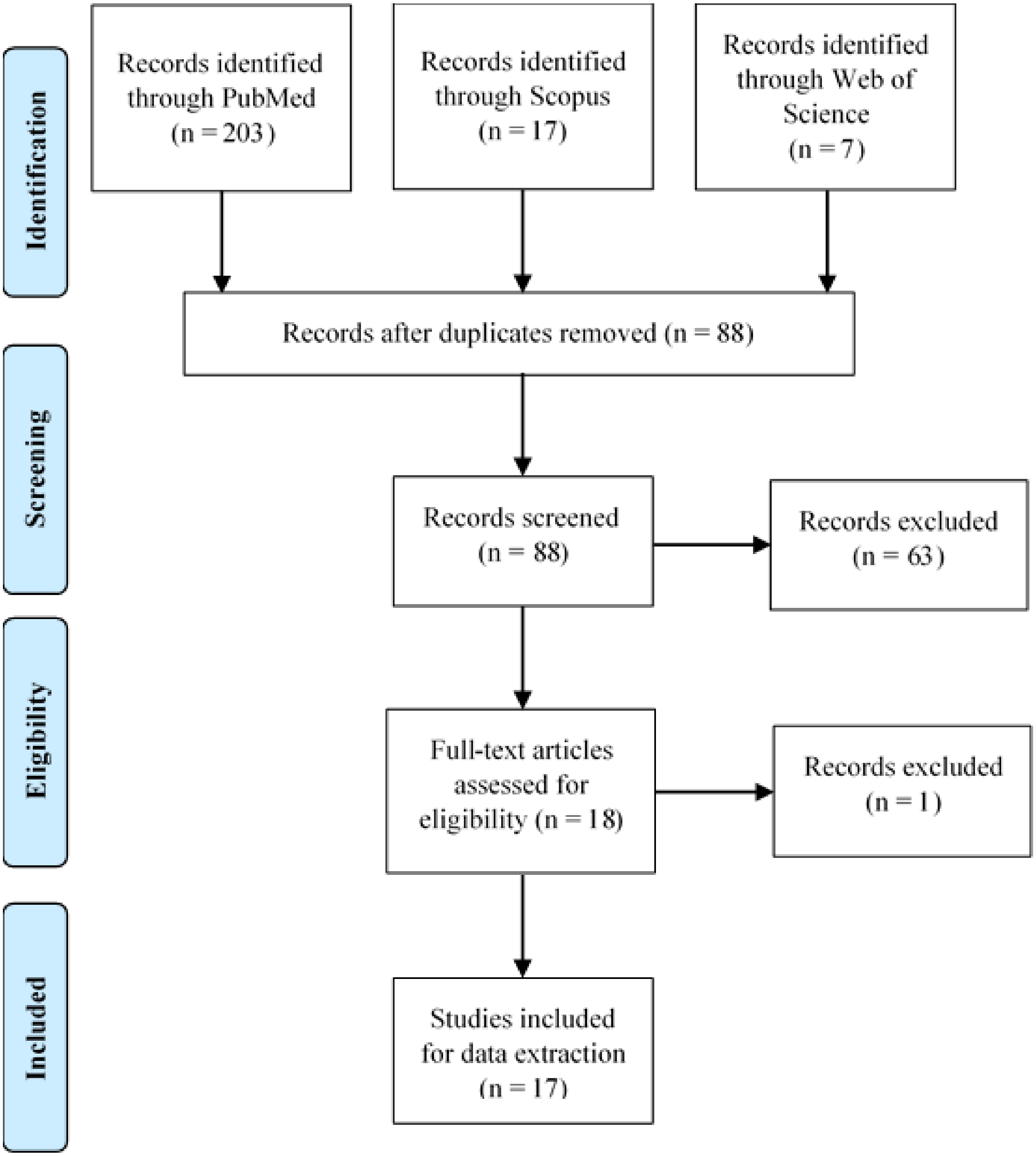


**Figure S1.** Preferred reporting items for systematic reviews and meta-analyses (PRISMA) flow diagram.
